# Correction

**DOI:** 10.1111/cas.15535

**Published:** 2022-11-03

In an article^1^ titled “Dahuang Fuzi Baijiang Decoction restricts progenitor to terminally exhausted T cell differentiation in colorectal cancer” by Xu, Yihua; Wang, Hao; Wang, Tao; Chen, Chunhui; Sun, Ruibo; Yao, Wanyu; Ma, Ye; Zhang, Qingyuan; Wu, Liyi; Zeng, Shanmei; Sun, Xuegang, the following errors were published:

In Figure 5, the label of “DFB” in Figure 5C should be modified to “CCR2[R]”, the correct figure is presented below.
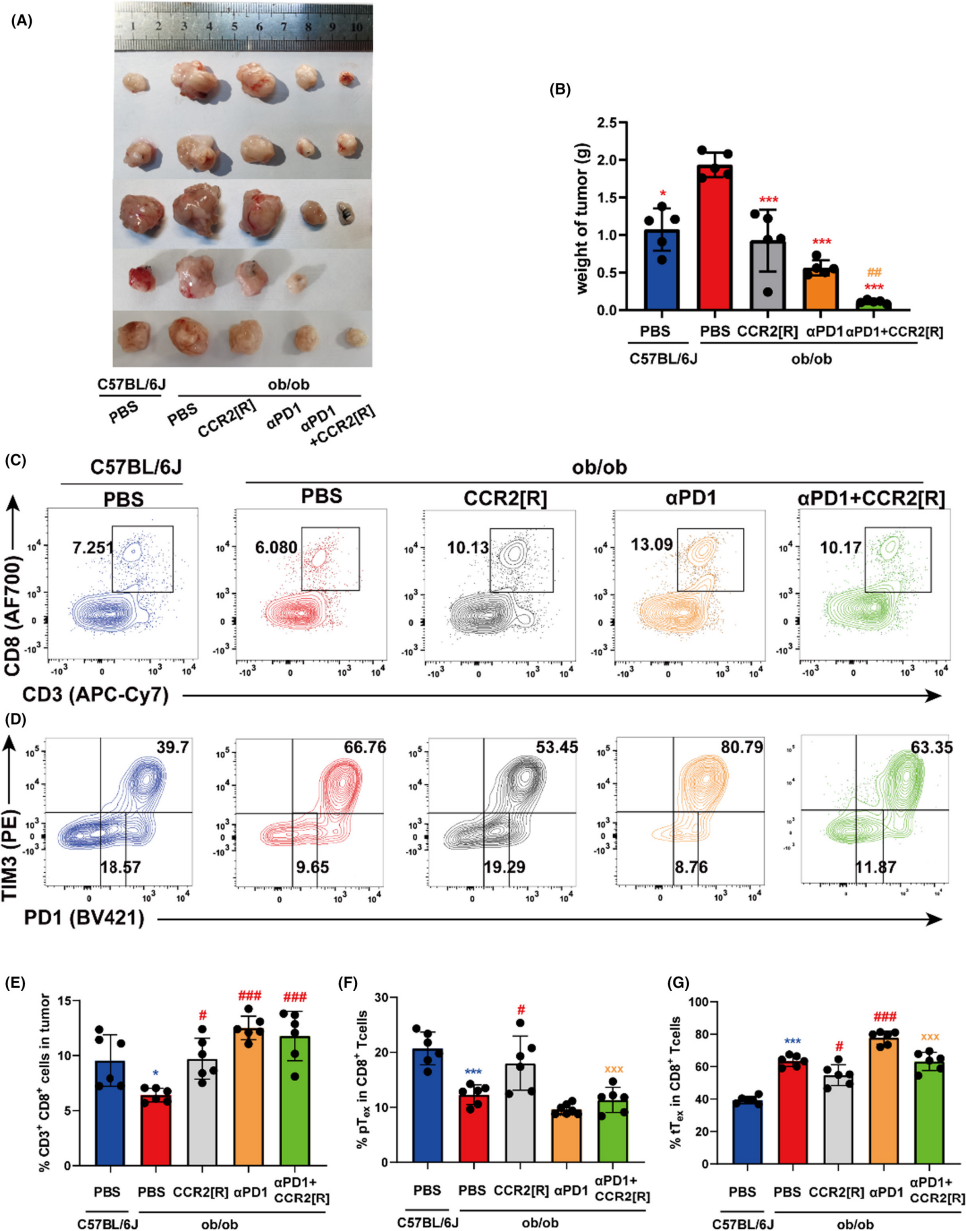


The authors apologize for the error.
